# Capsule Enlargement in *Cryptococcus neoformans* Is Dependent on Mitochondrial Activity

**DOI:** 10.3389/fmicb.2017.01423

**Published:** 2017-07-31

**Authors:** Nuria Trevijano-Contador, Suelen A. Rossi, Elisabete Alves, Santiago Landín-Ferreiroa, Oscar Zaragoza

**Affiliations:** Mycology Reference Laboratory, National Centre for Microbiology, Instituto de Salud Carlos III Madrid, Spain

**Keywords:** *Cryptococcus neoformans*, capsule enlargement, mitochondria, reactive oxygen species, mitochondrial membrane potential

## Abstract

*Cryptococcus neoformans* is an environmental encapsulated yeast that behaves as an opportunistic pathogen in immunocompromised individuals. The capsule is the main virulence factor of this pathogen. This structure is highly dynamic, and it can change its size and structure according to the environmental conditions. During infection, *C. neoformans* significantly enlarges the size of the capsule by the addition of new polysaccharide. It is believed that capsule growth is an energy-cost process, but this aspect has never been addressed. In this work, we have evaluated the role of mitochondrial activity on capsule growth using specific inhibitors of the electron respiratory chain. We observed that capsule growth was impaired in the presence of inhibitors of the respiratory chain as salicylhydroxamic acid or antimycin A. Furthermore, capsule growth correlated with an increase of the mitochondrial membrane potential and higher production of reactive oxygen species. Our results confirm that capsule growth depends on mitochondrial activity, and open new insights to understand the regulation of this process.

## Introduction

Cryptococcosis is a systemic infectious disease mainly caused by fungal species from the *Cryptococcus* genus. Its incidence increased in the twentieth century due to the emergence of HIV (Casadevall and Perfect, 1998). With the development of highly active antiretroviral therapy, the incidence has been controlled in developed countries, although the associated mortality remains high (Mirza et al., 2003; Friedman et al., 2005; Lortholary et al., 2006; Chottanapund et al., 2007; Dromer et al., 2007; Jongwutiwes et al., 2007). *Cryptococcus neoformans* is the species with the higher prevalence among HIV^+^ patients. Inhalation of infectious particles initiates a pulmonary infection (Casadevall and Perfect, 1998; Giles et al., 2009; Velagapudi et al., 2009; Botts and Hull, 2010; Heitman et al., 2011). Although the infection is normally controlled by immunocompetent hosts, the yeasts can proliferate and disseminate to the brain in patients with altered immune response, especially HIV^+^ and individuals who have suffered organ transplantation (Casadevall and Perfect, 1998; Singh et al., 2008). In these patients, it can cause meningoencephalitis, which has associated a high mortality if it is not diagnosed and treated effectively (Casadevall and Perfect, 1998). The impact of the disease is particularly significant in developing countries, where it has been estimated that it causes more than 650,000 deaths per year (Park et al., 2009).

The main phenotypic feature of *C. neoformans* is a capsule that surrounds the cell body (Benham, 1935) which confers physical, biochemical, and immunological properties to this microorganism. For this reason, the capsule has been the object of a great number of studies that have established their role as an important virulence factor (Chang and Kwon-Chung, 1994; Zaragoza et al., 2009; O’Meara and Alspaugh, 2012). The capsule of *C. neoformans* is composed of highly hydrophilic polysaccharide with a water content of 99% of its total weight (Maxson et al., 2007a). The capsule contains two main types of polysaccharides: glucuronoxylomannan and glucuronoxylomannogalactan, and a small proportion of mannoproteins (Evans and Mehl, 1951; Bhattacharjee et al., 1978, 1984; Cherniak et al., 1980, 1982; Murphy et al., 1988; Zaragoza et al., 2009). The capsular polysaccharides are released to the extracellular medium (exopolysaccharide) in vesicles, although this polysaccharide has different structure and antigenic properties than the one attached to the capsule (Rodrigues et al., 2007; Frases et al., 2008). The capsule and exopolysaccharides play an important role during infection because they have detrimental effects on the host (Vecchiarelli, 2000; McFadden and Casadevall, 2001; Zaragoza et al., 2009). Among others, the capsule inhibits the phagocytosis of the pathogen, blocks the migration and differentiation of lymphocytes, decreases antibodies production and induces apoptosis (Mitchell and Friedman, 1972; Murphy and Cozad, 1972; Lipovsky et al., 2000; Ellerbroek et al., 2002; Monari et al., 2008). These properties contribute to immune evasion. In addition, *C. neoformans* is an opportunistic intracellular pathogen, and the capsule has also an important role in the survival of the yeasts inside phagocytic cells (Steenbergen et al., 2001; Tucker and Casadevall, 2002; Johnston and May, 2013).

The size of the capsule is not constant, and during infection it increases significantly (Feldmesser et al., 2001). Capsule enlargement can be easily reproduced *in vitro* by incubation in low iron medium, enriched CO_2_ environment, mammalian serum, mannitol, or diluted nutrients at basic pH (Granger et al., 1985; Vartivarian et al., 1993; Zaragoza et al., 2003; Zaragoza and Casadevall, 2004; Guimaraes et al., 2010). Capsule growth occurs also in the presence of macrophage and amoeba extracts (Chrisman et al., 2010). In this sense, capsule growth confers resistance to stress conditions, such as reactive oxygen species (ROS) and antifungals so this process is presumably important to evade killing by phagocytic cells (Zaragoza et al., 2008).

**FIGURE 1:**
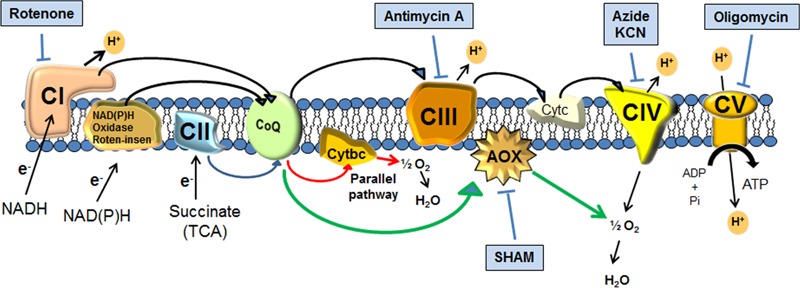
Scheme of the mitochondrial respiratory chain (modified from Alonso-Monge et al., 2009) and of the different electron transfer pathways: classical, alternative rotenone-insensitive, parallel (red arrows) and alternative oxidases (green arrows). The scheme also represents the target of the main respiratory inhibitors.

Capsule enlargement requires the accumulation of a large amount of new polysaccharide that requires the action of different enzymes that link the different monosaccharides by glycosidic bonds to yield a polysaccharide of a higher molecular weight (Pierini and Doering, 2001; Zaragoza and Casadevall, 2006; Doering, 2009; Frases et al., 2009; O’Meara and Alspaugh, 2012). It has been estimated that the increase in cell weight due to the capsule growth is about 20% of the total weight of the cells, and this process occurs in a few hours (Maxson et al., 2007b). For this reason, it is believed that this process is energetically costly for the cell. This is an important aspect, because capsule growth normally occurs in conditions of nutrient limitation. However, the metabolic changes and the molecular mechanisms that regulate capsule growth are not fully characterized, although several signaling pathways (cAMP and Hog1) and transcription factors (such as Cir1, Nrg1, Ada2, and Gat201) are required for capsule enlargement (Alspaugh et al., 1997; Zaragoza et al., 2003; Cramer et al., 2006; Liu et al., 2008; Haynes et al., 2011). In eukaryotes, energy is obtained mainly from the production of ATP during mitochondrial respiration. In animals, the electron transfer during respiration occurs through the complexes of the inner mitochondrial membrane known as classical pathway. Higher plants, algae, and fungi possess different electron transfer pathways In addition to the electron transfer from NADH to complex I (rotenone sensitive), there are also other NAD(P)H oxidases (rotenone-insensitive) which do not contribute to proton efflux to the intermembrane space (Rasmusson and Møller, 1991; Kerscher, 2000). Fungi and plants can use electrons of the respiratory chain through the alternative oxidases (AOX) pathway (Vanlerberghe, 2013). Finally, in some *Candida* spp., such as *Candida albicans* and *Candida parapsilosis*, a parallel pathway that uses parallel cytochrome *c* proteins has been described (see Milani et al., 2001; Alonso-Monge et al., 2009; **Figure 1**). Since capsule growth is a process that requires the synthesis and accumulation of new polysaccharide, we argued that it is energetically costly for the cell. For this reason, we decided to investigate the role of the mitochondria on capsule growth. We demonstrate that inhibition of mitochondrial activity by blocking different complexes impairs capsule enlargement, and that this process is associated with changes in mitochondrial potential. Our data suggest that under the stress conditions that lead to capsule growth (such as nutrient-limitation), part of cellular energy is invested in capsule enlargement, which highlights the importance of this structure for the survival in the yeast.

## Materials and Methods

### Strains, Media, and Growth Conditions

All experiments were performed with *C. neoformans* var. *grubii* strain H99 (Perfect et al., 1980). The yeasts were routinely grown in liquid Sabouraud medium at 30°C with moderate shaking (150 rpm). For capsule induction, the cells were transferred to capsule inducing media (10% Sabouraud buffered at pH 7.3 with 50 mM MOPS) for 0, 3, 6, and 24 h (Zaragoza and Casadevall, 2004).

### Metabolic Inhibitors

We have tested the following compound which inhibit specific complexes of electron transport chain: antimycin A (Sigma–Aldrich, stock 1.82 mM in ethanol 96%), oligomycin (Sigma–Aldrich, stock 1.26 mM, in ethanol), potassium cyanide (KCN, Sigma–Aldrich, stock 80 mM in distilled water), salicylhydroxamic acid (SHAM, Sigma–Aldrich, stock 160 mM in ethanol), rotenone (Sigma–Aldrich, stock 100 mM in DMSO), and sodium azide (Sigma–Aldrich, stock 150 mM in distilled water). Rotenone inhibits complex I of electron transport chain, antimycin A inhibits complex III, SHAM inhibits alternative cytochrome oxidative pathway, KCN and sodium azide the complex IV and Oligomycin inhibits complex V.

### Growth Curves in the Presence of Mitochondrial Inhibitors

To perform growth curves, the cells were harvested with centrifugation at 3,000 *g* for 3 min and washed three times with phosphate-buffered saline (PBS). Cellular suspensions were prepared at 2 × 10^5^ cells/mL and inoculated in triplicate in a 96-well plate. Serial 1:2 dilutions of the inhibitor stocks were performed with PBS at the following concentrations: KCN from 20 to 2.5 mM, antimycin A from 3.6 to 0.22 μM, Oligomycin from 15 to 0.94 μM, SHAM from 4 to 0.47 mM, rotenone from 250 to 15.6 μM and sodium azide from 7.6 to 0.003 mM. Seventy-five microliters of the inhibitors were mixed with the same volume of the yeast cells suspensions prepared in Sabouraud 2× in 96-well plates which was incubated at 30°C with moderate shaking in a spectrophotometer iEMS (Thermo Fisher Scientific). Optical density was measured at 540 nm every hour for 48 h. Data were analyzed with GraphPad Prism 5.

We also tested if the inhibitors had fungistatic or fungicidal effect on *C. neoformans*. For this purpose, after the 48 h of incubation in the 96-well plates as described above, 10 μL from the wells of microdilution plates were placed on Sabouraud agar plates. The growth of the yeast on the agar plates was evaluated after 48 h of incubation at 30°C.

### Induction of Capsule Growth in the Presence of Mitochondrial Inhibitors

Cells from H99 strain were incubated capsule induction medium or Sabouraud at 10^6^ cells/mL in the presence of different mitochondrial inhibitors (from a 100× stock solution). Parallel samples with the same volume of the corresponding solvent were carried out as control. The flasks were incubated overnight at 37°C. To observe and measure the size of the capsule, 10 μL of a cell suspension were mixed with an India ink and observed in a Leica DMI 3000B microscope. To calculate capsule volume, the diameter of the whole cell and the cell body were each measured with Adobe Photoshop 7.0 and capsule volume was defined as the difference between the volume of the whole cell (yeast cell + capsule) and the volume of the cell body. Twenty cells were measured for each determination.

### Cellular Viability Measured by Flow Cytometry Assay

Cells were treated with mitochondrial inhibitors during capsule induction as described above. Then, the cells were collected by centrifugation (3,000 *g*, 3 min), washed twice with PBS and adjusted to 10^6^ cells/mL. An untreated sample was used as negative control. In parallel, a sample was placed a 60°C during 30 min as a control of heat-killed cells. Propidium iodide (Sigma-Aldrich, St. Louis) was added at 5 μg/mL and fluorescence of the dead cells was measured using a FacsCalibur cytometer (BD Biosciences, Woburn, MA, United States) in the FL3 channel. Data obtained were analyzed with the programs CellQuest (BD Biosciences) and FlowJo (Tree Star Inc, Ashland, OR, United States).

### Real-Time PCR

RNA extraction was performed using Trizol reagent (Ambion RNA by Life technologies) following the manufacturer’s recommendations with minor modifications. The cells were disrupted with glass beads using a FastPrep-24 (MP^TM^) for 5 min, alternating 20 s shaking with 1 min on ice. The RNAs were quantified using the Nanodrop 8000 Spectrophotometer (Thermo Fischer Scientific). RNA samples (0.1 μg/μL) were treated with DNase using the DNA-free^TM^ kit (Thermo Fisher Scientific). cDNAs were generated using the iScrip^TM^ cDNA synthesis Kit (Bio-Rad) following the manufacturer’s recommendations. The RT-PCR was performed using SsoAdvanced^TM^ Universal SYBR^R^ Green Supermix (Bio-Rad) using the *COX1* (cytochrome *c* oxidase subunit I encoding gene) specific primers (COX1F CTGGTATGACACTACACAAGATGCCTC and COX1R ACCA1GCTAGAACTGGGATACATAGGA) described in Alanio et al. (2011) in a total volume of 10 μL, in a Light Cycler^R^ 480. As control, 18s specific primers were used (18sF CCGTTGCTAGAGGTGAAATTCTTAG and 18sR ATCTAATCGTTTTTGATCCCCTAAC). The RT-PCR conditions were (95°C for 10 min and 40 cycles of amplification (95°C for 15 s, 58°C for 1 min). Differences in gene expression were calculated using the 2^ΔΔCt^ method (Pfaffl, 2004).

### Mitochondrial Membrane Potential Assay

Mitochondrial membrane potential was measured by flow cytometry using the dye Rhodamine 123 (Invitrogen), which are internalized and accumulated in the mitochondria by a process dependent on the mitochondrial membrane potential. Cells were harvested by centrifugation (3 min at 3,000 *g* at room temperature), washed twice with PBS and suspended in the capsule inducing media at 10^6^ cells/mL. Ten milliliters from this suspension were incubated for 3, 6, and 24 h at 37°C. The cells were suspended in sodium citrate buffer (50 mM, pH 5), containing 2% glucose, and the rhodamine 123 was added at a final concentration of 35 μM. Cells were incubated at room temperature for 30 min and fluorescence was visualized by microscopy. Then, the fluorescence intensity was quantified using flow cytometry using a FacsCalibur (BD Biosciences, Woburn, MA, United States) and data were analyzed with CellQuest (BD Biosciences) and FlowJo (Tree Star Inc, Ashland, OR, United States).

### Detection of ROS with Fluorescent Probes

The detection of ROS accumulation was performed with dihydrofluorescein diacetate (DHF, Sigma-Aldrich), which produces green fluorescence after attack by ROS. A suspension of 10^6^ cells/mL was prepared and incubated at 37°C for different times in Sabouraud or in capsule-inducing medium. In addition, AmB (1 μg/mL) was added to same samples as a control of ROS production (Sangalli-Leite et al., 2011). After each incubation time DHF was added at a final concentration of 40 μM and the samples were incubated a 37°C for 30 min. Then, the cells were fixed with *p*-formaldehyde 4% during 30 min at room temperature and were washed with PBS. The fluorescence was quantified using a FacsCalibur cytometer (BD Biosciences, Woburn, MA, United States).

### Statistics

The data was analyzed with GraphPad 5.0 software (GraphPad Software, La Jolla, CA, United States) and the Kolmogorov–Smirnov test was used to know the normality samples. ANOVA and *t*-test were used for normally distributed samples and Kruskal–Wallis and Mann–Whitney tests for samples considered not normal. Statistics difference was considered when *p* < 0.05.

## Results

### Effect of Inhibition of the Respiratory Chain on Cell Growth

**FIGURE 2:**
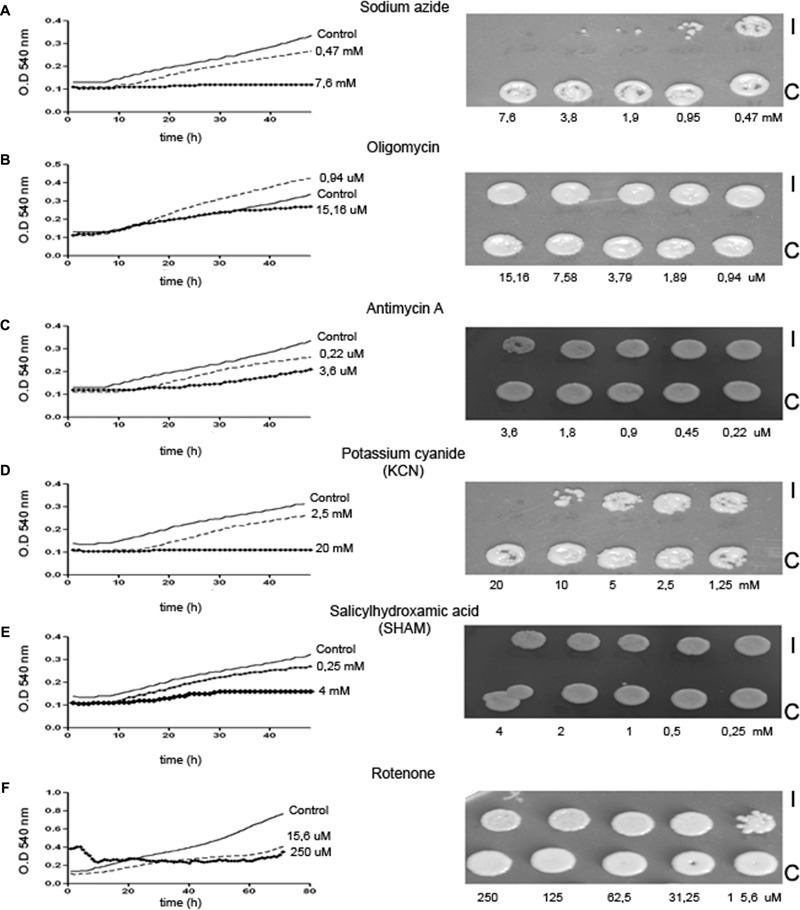
Effect of mitochondrial inhibitors on *Cryptococcus neoformans*. *C. neoformans* cells were cultured in liquid Sabouraud in presence of different metabolic inhibitors [sodium azide **(A)**, oligomycin **(B)**, antimycin A **(C)**, potassium cyanide **(D)**, salicylhydroxamic acid **(E)**, and rotenone **(F)**] in 96-well plates and growth curves were performed at 48 or 72 h at 30°C. After this incubation time, 10 μL from the wells incubated with the inhibitor (I) or control carrying only the same concentration of solvent (C) were placed on Sabouraud agar plates and incubated at 30°C during 48 h. After that time, photographs were taken (right panels).

To investigate to what extend capsule enlargement depends on the metabolic activity of the cell, we inhibited mitochondrial metabolism with different compounds and tested their effect on capsule production. First, we evaluated how these inhibitors affected the growth and viability of *C. neoformans* cells. We performed growth curves in Sabouraud liquid medium in presence of inhibitors for 48 h at 30°C to determine the minimal inhibitory concentration. All inhibitors caused a decrease in growth, which was dependent on the concentration (**Figure 2**, left part). The inhibition was observed from the lowest concentration used for each inhibitor, with the exception of oligomycin where all the concentrations evaluated grew equal or more than the control. In addition, no growth was observed at the highest concentrations of sodium azide and KCN.

Then, we wanted to establish if the mitochondrial inhibitors had fungicidal or fungistatic effect on *C. neoformans*. For this purpose, we placed an aliquot of yeasts from the wells in which the inhibitors blocked growth from the 96-well plates in Sabouraud agar plates and incubated them for 24 h at 30°C. We found that antimycin A, SHAM, and rotenone inhibited growth but did not kill the cells. In contrast, cyanide and sodium azide were fungicidal (**Figure 2**, right panels and **Table 1**).

**Table 1:** Summary of the effect of different mitochondrial inhibitors on *C. neoformans*.

Inhibitor	Solvent	Inhibiting concentration	Antifungal effect
Antimycin A	Ethanol (96%)	0.22 μM	Fungistatic
Oligomycin	H_2_O	–	No effect
Potassium cyanide	Ethanol (96%)	5 mM	Fungicidal
Salicylhydroxamic acid	Ethanol (96%)	0.5 mM	Fungistatic
Rotenone	H_2_O	15.6 μM	Fungistatic
Sodium azide	DMSO (100%)	0.95 mM	Fungicidal

*The table represents the minimal inhibiting concentration and whether the inhibitors show fungicidal or fungistatic effect.*

### Effect of Specific Inhibitors of the Electron Respiratory Chain on Capsule Enlargement

**FIGURE 3:**
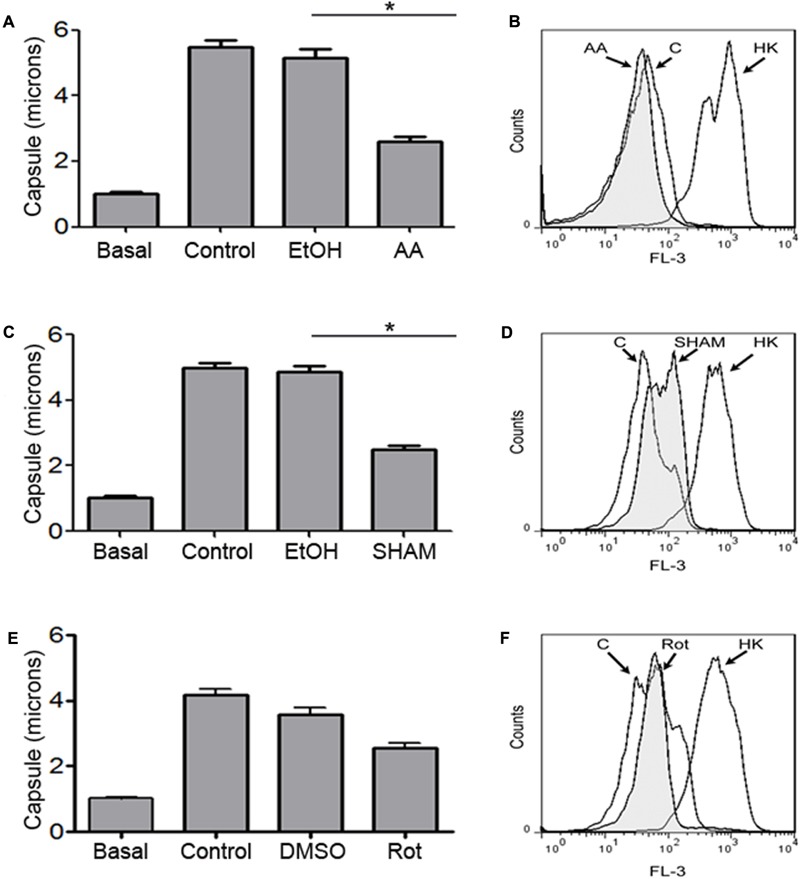
Effect of mitochondrial inhibitors during capsule induction. Cells from H99 strain were grown in Sabouraud liquid medium at 30°C (basal) and then transferred to 10% Sabouraud buffered at pH 7.3 with 50 mM MOPS in the presence of the highest concentration of antimycin A (3.6 μM) **(A,B)**, SHAM (4 mM) **(C,D)**, and rotenone (250 μM) **(E,F)** for 18 h. In each case, control samples incubated only with the solvent (EtOH or DMSO) or without any solvent (control) were carried out in parallel. After the incubation time, capsule size **(A,C,E)** was measured by suspending the cells in India ink as described in Section “Materials and Methods.” Asterisks indicate significant differences (*p* < 0.05). In parallel, cellular viability was determined by measuring the uptake of propidium iodide by dead cells by flow cytometry **(B,D,F)**. Heat-killed cells (HK) were included as positive controls. The experiment was performed in triplicate.

Next, we evaluated the role of mitochondrial activity on capsule growth. We chose those inhibitors that had fungistatic effect on *C. neoformans*. First, cells were grown in liquid Sabouraud at 30°C overnight and transferred to capsule inducing medium with the highest concentrations of the mitochondrial inhibitors. We found that these inhibitors impaired capsule enlargement compared with the control (**Figures 3A,C,E**). We also analyzed viability by flow cytometry during the capsule induction to discard that lack of capsule induction was due to cell death. So we measured the uptake of propidium iodide by the cells as an indicator of cell death. As show in **Figures 3B,D,F**, the cells did not become permeable to PI, indicating that none of the inhibitors produced cell death. Our results showed that inhibition of these mitochondrial complexes inhibits capsule enlargement.

### Changes in Mitochondrial Membrane Potential during Capsule Growth

**FIGURE 4:**
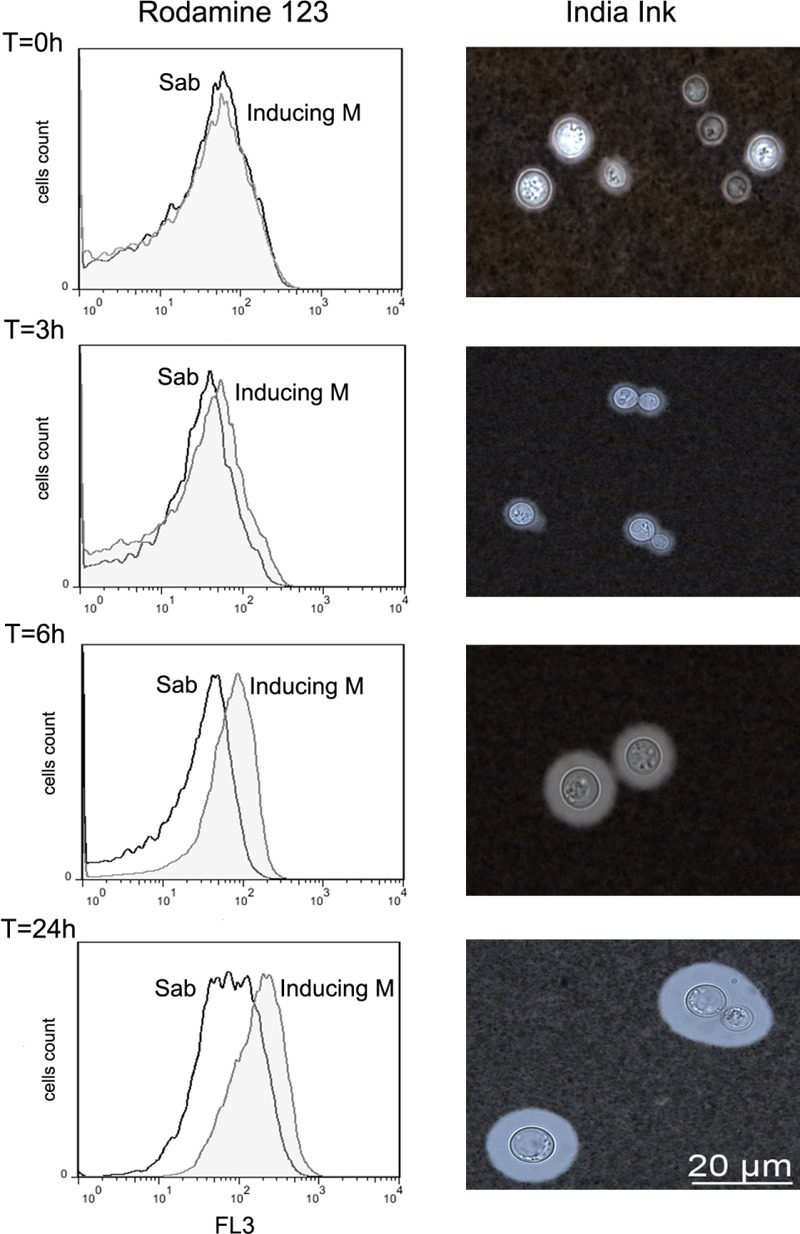
Evaluation of mitochondrial membrane potential during capsular growth. Cells from H99 strain were incubated in capsule induction medium or Sabouraud for 0, 3, 6, and 24 h, and after each time, rhodamine 123 was added (see Materials and Methods). The uptake of this probe was analyzed by flow cytometry (left panel). The graph represents the results of a representative experiment, which was performed three different times. Cells in Sabouraud (Sab) are represented with histograms in black line and the cells in capsule inducing medium (MOPS) are represented in gray histograms. Representative cells from each time in the capsule inducing medium are shown in the right panels.

We examined if during capsule enlargement there were any changes in mitochondrial activity. Therefore, we evaluated the mitochondrial membrane potential as an indicator of the functionality of the mitochondria using the fluorescent probe Rhodamine 123 (Alonso-Monge et al., 2009), whose accumulation in the mitochondria depends of the membrane potential. We observed that cells accumulated more Rhodamine 123 when they were incubated in inducing medium compared to the same cells in Sabouraud (**Figure 4**). This result suggests that during capsule induction there was an increase in the mitochondrial membrane potential.

### Expression of *COX1*

**FIGURE 5:**
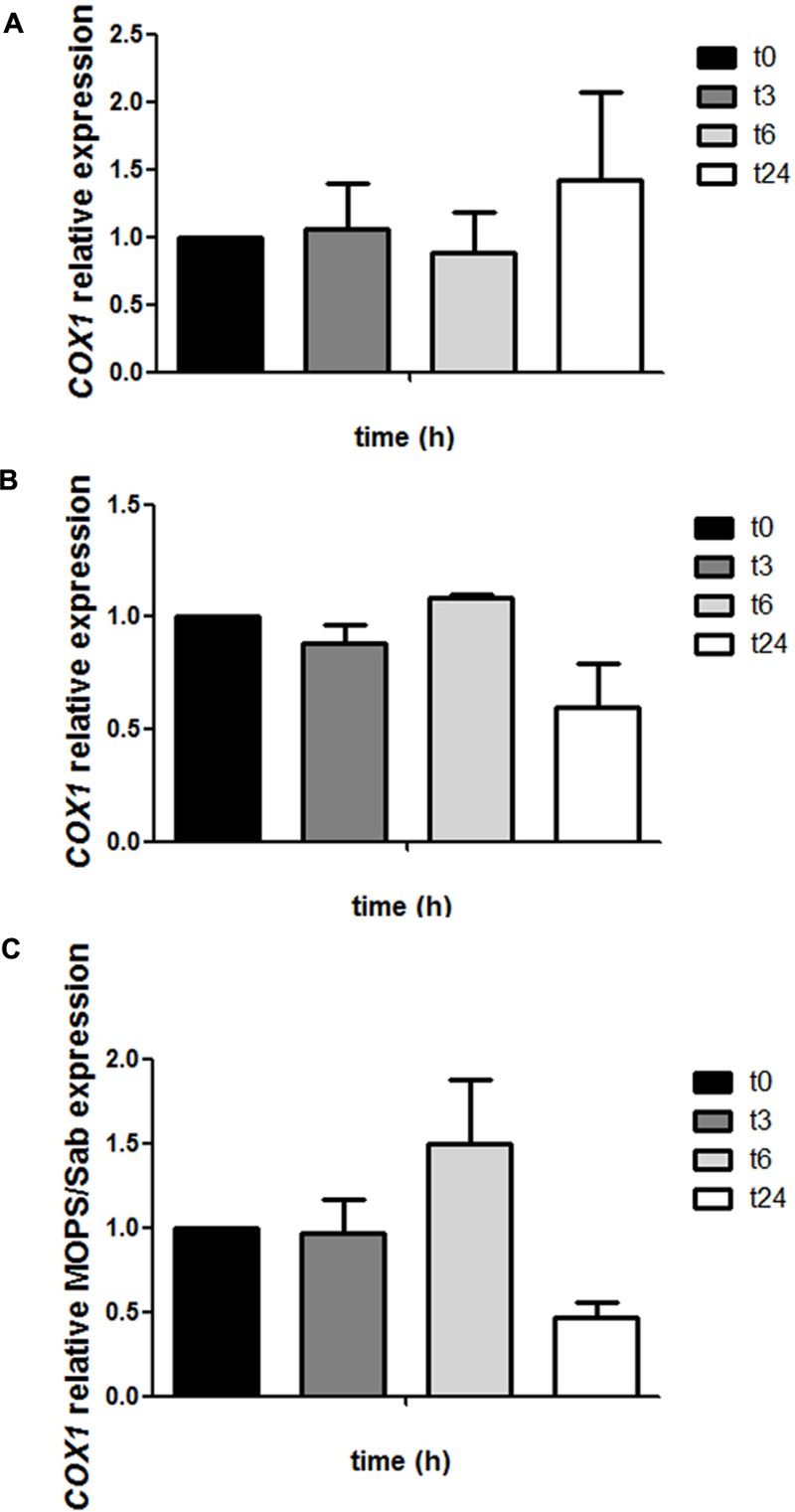
Analysis of *COX1* expression during capsule induction. Cells from H99 strain were grown in Sabouraud and capsule inducing medium at 37°C and RNA was isolated from samples were collected at different times (0, 3, 6, and 24 h). cDNAs were obtained and relative expression of the *COX1* gene was measured by real-time PCR as described in Section “Materials and Methods.” *COX1* gene expression at different time points in Sabouraud medium **(A)** and capsule inducing medium **(B)**. Then, a relative fold change comparing at each time point the difference between the capsule inducing medium and Sabouraud was plotted in **(C)**. This experiment was performed in triplicate.

*COX1* encodes cytochrome *c* oxidase, which is a mitochondrial enzyme involved in respiration and thus energy production in *C. neoformans*. We evaluated the expression of the *COX1* gene by real-time PCR during capsule induction or cells maintained in Sabouraud (**Figure 5**). We found that mRNA levels of *COX1* tended to be higher during incubation in capsule growth than in Sabouraud, being the difference more noticeable after 6 h of incubation. Interestingly, this effect was not due to an increase of the expression of the gene in our inducing conditions, but rather to a gradual decrease in the rich medium while COX1 expression remained constant in capsule growth conditions.

### Detection of ROS during Capsule Induction

**FIGURE 6:**
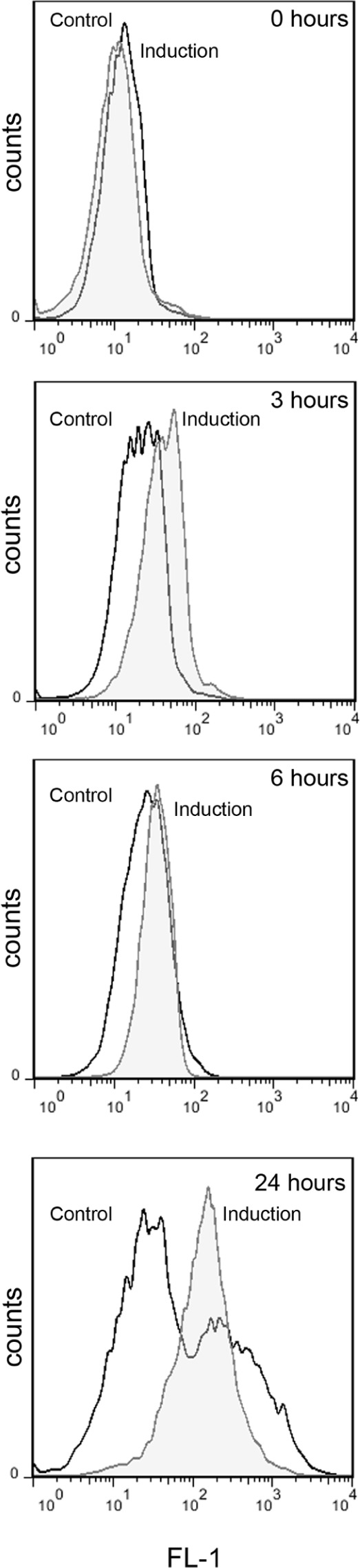
Production of ROS during capsule induction. Cells from H99 strain were grown in Sabouraud overnight then transfer to the same medium (control, black line) or capsule induction medium (induction, gray filled histogram). The production of ROS was evaluated at time 0, 3, 6, and 24 h by flow cytometry as described in Section “Materials and Methods.” As positive control we used samples in the presence of AmB (1 μg/mL). The graph represents the results of a representative experiment, which was performed three different times.

ROS are produced in the mitochondria as a result of the oxidative metabolism. We argued that if during capsule growth there are changes in mitochondrial activity, there might be also a differential accumulation of ROS compared to capsule non-inducing conditions. In this experiment, we observed that the ROS production increased during the induction medium over time. The ROS accumulation started after 3 h and was more evident after 24 h of incubation in comparison with the cells grown in Sabouraud medium (**Figure 6**). In all experiments, we included AmB (1 μg/mL) as a positive control for (data not shown).

## Discussion

The polysaccharide capsule is the most characteristic feature of the fungal pathogen *C. neoformans*, and it is its main virulence factor (Zaragoza et al., 2009). For this reason, the synthesis and factors that regulate its size and structure have been largely studied (Pierini and Doering, 2001; McFadden et al., 2006; Zaragoza et al., 2006; Maxson et al., 2007b). However, some of the key aspects of the capsule still remain to be elucidated. For example, the molecular mechanisms that trigger capsule enlargement and cellular changes associated to this transition are unknown. Capsule growth occurs by synthesis and addition of new polysaccharide (Zaragoza et al., 2006), and is dependent on protein synthesis (Doering, 2009). The increase in size elicited by *C. neoformans* pose a dramatic change for the cell. For example, it has been shown that during this process, the cell increases its dry mass around 20% in a few hours (Maxson et al., 2007b), and the total volume of the capsule poses around 90% of the total volume of the cell. Most of the media that induce capsule growth are limited in the concentration of nutrients (i.e., serum, DME, and CO_2_ atmosphere, low iron medium). For this reason, we found intriguing that in these conditions there is such an accumulation of new polysaccharide on the capsule. Due to the magnitude of the change, we argued that capsule enlargement is a process that requires a high investment of the available energy in the cell. Since *C. neoformans* is a respiratory yeast, energy is mainly produced at the mitochondria. Therefore, we investigated to which extent capsule enlargement requires the correct functioning of the electron chain. Fungi present some differences in the electron transfer in the mitochondria compared to mammals. In particular, oxygen is reduced through several pathways. In addition to the classical respiratory chain through the mitochondrial complexes, electrons are transferred to oxygen through AOX and cytochrome *c* (Kerscher, 2000; Alonso-Monge et al., 2009). We found that blocking the classical respiratory chain or the pathway of the AOX is enough to inhibit capsule growth. Our data indicates that mitochondria play an important role in capsule synthesis. Furthermore, the fact that inhibition of the AOX pathways with SHAM (which does not affect the classical respiratory chain) has a similar effect indicates that mitochondrial unbalance drastically affects cellular metabolism and capsule growth. At the moment, we do not know how alterations of AOX pathway exactly affect the mitochondrial respiratory chain (classical pathway) or how the cells exactly obtain the energy in these conditions. AOXs have been postulated to play a homeostatic role in the electron transfer in the mitochondria (Vanlerberghe, 2013), so it is possible that inhibition of this pathway there is an accumulation of ROS in the cells that cause deleterious effects effect in the cells. It is also possible that inhibition of AOX pathway results in a higher electron flux through the parallel pathway, which does not contribute ATP production, which could also limit the available energy of the cell and impair capsule enlargement. In our conditions, capsule growth occurs during nutrient starvation. In these conditions, we argue that the cells dedicate an important amount of energy in the adaptation responses that are required to survive in the new conditions. But our results suggest that capsule indicate that capsule growth can be considered a stress response which requires part of the energy produced by the cell. This is supported by the fact that capsule growth requires the synthesis of a significant amount of new polysaccharide.

We also confirmed that capsule enlargement correlates with mitochondrial changes, such as higher mRNA levels of *COX1* and mitochondrial membrane potential. These data suggest that during this process, there is higher rate of electron transfer through the respiratory complex compared to basal conditions that results in a higher concentration of protons in the interperiplasmic space. The increase in mitochondrial activity also correlates with higher basal levels of ROS detected during capsule growth. The results from this work could should be complemented with other techniques that measure the respiration rate and oxygen consumption (with O_2_ detectors or Warburg respirometers). This work opens future research lines that will highlight the importance of specific metabolic pathways (such as the importance of the carbon and nitrogen source) on capsule growth. We also believe that future studies that determine the exact energy cost and energy flow during capsule growth are warranted.

We found that oligomycin did not have any effect on *C. neoformans*, which is in agreement with previous findings in the literature (Hua et al., 2000). This compound is an inhibitor or ATP synthase (complex V). At the moment, it is not known why this inhibitor is not active against *C. neoformans*, although it is possible that this yeast possesses a protein with some variants that makes the cells resistant.

Capsule growth is a key phenotype that determines the interaction of *C. neoformans* with the host and is a particular feature that is not expressed by other fungal pathogens. Several genes required for this process have been identified, such as *ADA2* and *GAT201* (Liu et al., 2008; Haynes et al., 2011). However, there are many aspects about this process that remain unknown. For example, capsule growth occurs in the G1 phase of cell cycle (Garcia-Rodas et al., 2014), but the link between the addition of polysaccharide to the capsule and cell cycle regulation has not been determined. Another intriguing aspect is the regulation of capsule size, and more in particular, why the size of the capsule does not increase more than a certain size (Zaragoza et al., 2006). In this work, we have addressed a novel aspect of capsule growth, which is the relationship with the cell metabolism. The availability of energy and mitochondrial functioning is a key process in the cell to ensure the correct response of the cell to environmental stimuli and challenges, and many stress factors induce changes in mitochondrial metabolism and ROS accumulation. We hypothesize that capsule enlargement is an important process to protect against stress factors, and for this reason, a significant amount of the energy of the cell is invested in increasing capsule size.

Our results open new questions on the field, such as what the metabolic cost of capsule enlargement and how the cells rearrange their metabolism during these conditions.

## Author Contributions

NT-C, SR, EA, and SL-F participated in experimental design, development of the experiments, and preparation of the manuscript. OZ participated in experimental planning and writing of the manuscript.

## Conflict of Interest Statement

The authors declare that the research was conducted in the absence of any commercial or financial relationships that could be construed as a potential conflict of interest.
